# Identification of sapovirus GV.2, astrovirus VA3 and novel anelloviruses in serum from patients with acute hepatitis of unknown aetiology

**DOI:** 10.1371/journal.pone.0185911

**Published:** 2017-10-05

**Authors:** Eloy Gonzales-Gustavson, N. Timoneda, X. Fernandez-Cassi, A. Caballero, J. F. Abril, M. Buti, F. Rodriguez-Frias, R. Girones

**Affiliations:** 1 Laboratory of Virus Contaminants of Water and Food, Department of Genetics, Microbiology and Statistics, Faculty of Biology, University of Barcelona, Barcelona, Catalonia, Spain; 2 Computational Genomics Lab, Department of Genetics, Microbiology and Statistics, Faculty of Biology, University of Barcelona, Barcelona, Catalonia, Spain; 3 Institut de Biomedicina de la Universitat de Barcelona (IBUB), Barcelona, Catalonia, Spain; 4 Hospital Universitari Vall d’Hebron and CIBEREHD del Instituto Carlos III, Barcelona, Catalonia, Spain; Defence Research Laboratory, INDIA

## Abstract

Hepatitis is a general term meaning inflammation of the liver, which can be caused by a variety of viruses. However, a substantial number of cases remain with unknown aetiology. We analysed the serum of patients with clinical signs of hepatitis using a metagenomics approach to characterize their viral species composition. Four pools of patients with hepatitis without identified aetiological agents were evaluated. Additionally, one pool of patients with hepatitis E (HEV) and pools of healthy volunteers were included as controls. A high diversity of anelloviruses, including novel sequences, was found in pools from patients with hepatitis of unknown aetiology. Moreover, viruses recently associated with gastroenteritis as sapovirus GV.2 and astrovirus VA3 were also detected only in those pools. Besides, most of the HEV genome was recovered from the HEV pool. Finally, GB virus C and human endogenous retrovirus were found in the HEV and healthy pools. Our study provides an overview of the virome in serum from hepatitis patients suggesting a potential role of these viruses not previously described in cases of hepatitis. However, further epidemiologic studies are necessary to confirm their contribution to the development of hepatitis.

## Introduction

Hepatitis is a general term meaning inflammation of the liver and can be caused by a variety of viruses, such as hepatitis A, B, C, D and E [1]. Infectious agents such as bacteria, fungi or parasites, as well as non-infectious agents such as alcohol, drugs or autoimmune diseases, may cause hepatitis too. According to the estimates of the Global Burden of Disease study, viral hepatitis is responsible for approximately 1.5 million deaths each year, which is comparable to the number of annual deaths from HIV/AIDS (1.3 million), malaria and tuberculosis (TB) (0.9 million and 1.3 million, respectively) [2].

Viral hepatitis is still one of the key causes of acute liver failure (ALF) in the world. ALF is a devastating clinical syndrome associated with high mortality in the absence of immediate care, specific treatment or liver transplantation [3]. Globally, hepatitis A, B and E infections are probably responsible for the majority of ALF cases. However, despite significant progress in the diagnosis and treatment of hepatitis, in a considerable number of patients, the aetiological agents remain unknown. Previous studies have found that between 3.8% and 33.9% of hospital inpatients with acute hepatitis had non-A-E-hepatitis [4–8]. Additionally, 10% of patients with ALF had non-A-E hepatitis [9].

Therapeutic trials using interferon-α to treat hepatitis of unknown aetiology have consistently resulted in response rates of approximately 50%, indicating a virological aetiology [10]. This evidence suggests that other viruses may be responsible for hepatitis. As a result, new viruses, including a *Flaviviridae* GB virus type C (GBV-C) [11] and *Anelloviridae* TTV and SEN virus [12], have been reported in recent years to be associated with hepatitis. However, epidemiological data failed to confirm a causative role for those viruses in the development of hepatitis, and a high percentage of individuals infected by them were found to be healthy carriers [13,14]. Recent investigation has shown that other viral infections such as cytomegalovirus and Epstein Barr virus may mimic viral hepatitis [15]. Less frequently, hepatitis may be present in people with herpes simplex virus [16], parvovirus B19 [17], and adenoviruses 1, 2, 5, 12 and 32 [18,19].

Epidemiologic information related to non-A-E hepatitis is scarce. In a study by Delic et al. 2010, analysing 408 patients with acute hepatitis, history of blood transfusion, drug use or other parenteral exposure were not associated with the onset of illness [7], suggesting that if the viral nature of non-A-E hepatitis is proven, it should spread primarily by non-parenteral means. Moreover, some patients diagnosed with acute non-A-E hepatitis show biochemical features at admission similar to those associated with other viral hepatitis. Apparently, acute non-A-E hepatitis is distributed worldwide, and progression to chronicity was observed in approximately 9% of patients [7,20].

The cause of acute non-A-E hepatitis remains unknown. It seems likely that another as-yet-unidentified infectious agent(s) exists [20]. Recent rapid progress in sequencing technologies and associated bioinformatics methodologies has enabled a more in-depth view of the structure and functioning of viral communities, supporting the characterization of emerging viruses [21]. With the advent of metagenomics studies, our knowledge of the different components and the complexity of the microbiome greatly expanded. The eukaryotic virome comprises viruses infecting the host, endogenous viral elements, and viruses associated with other eukaryotic components of the ingesta [22].

In this study, next-generation sequencing (NGS) was used to identify viruses in serum samples from patients suffering from acute hepatitis signs. For that purpose, the viromes in the serum of patients with Non-A-E hepatitis were analysed and the results were compared with the viromes from patients with acute hepatitis E (positive controls) and healthy patients (negative controls).

## Materials and methods

### Serum samples

A total number of 42 serum samples were collected from patients with acute viral hepatitis from the Vall d’Hebron Hospital, Barcelona, Spain. The clinical diagnosis of acute viral hepatitis was based on the lack of previous history of chronic liver disease, a rise in serum aminotransferase (AST, ALT) activity of at least 200 IU/L, high values of total (TB) and direct bilirubin (DB) and exclusion of other causes of liver disease such as hepatitis A (Ig-M negative), hepatitis B (surface-antigen-HBsAg- and anti-core antibodies-anti-HBc-negative-), hepatitis C (anti-VHC-negative) and hepatitis E (HEV) (IgG, IgM and RT-PCR, all negatives). Of the 32 patients with acute hepatitis of unknown aetiology, 19 were male, and 13 were female, with ages ranging from one to 92 years old. Eight of those patients were diagnosed with an autoimmune or immunosuppressed (Ai+ImSP) condition. Additionally, serum samples from 10 patients—positive for HEV by nested RT-PCR—were included as positive controls. In addition, serum samples from 20 healthy volunteers were also evaluated.

The serum samples were pooled according to the following criteria. Patients with acute hepatitis were grouped into five pools: male pool A (8 samples, age range from 1 to 44), male pool B (8 samples, age range from 45 to 78), a female pool (8 samples, age range from 6 to 92), and an Ai+ImSP pool (8 samples, age range from 2 to 84) that included patients with the Ai+ImSP condition. Finally, a pool of HEV RNA-positive patients (10 samples, age range from 6 to 84) was included. Healthy volunteers’ serum samples were grouped in two pools and evaluated in duplicate: Healthy A1 and A2 pools, with 10 females (age range between 27 and 63), and Healthy B1 and B2 pools, with 2 males and 8 females (age range between 26 and 58).

### Sample preparation

Serum samples were kept at -80°C prior to the metagenomics analysis protocol. Pools were prepared with the corresponding serum samples to achieve an initial volume of 500 μL. Briefly, the pools were first filtered through a pore size of 0.45 μm (Millipore Corp., Billerica, MA, USA) to remove cellular debris, ultracentrifuged at 100,000 × g for 90 min at 4°C and re-suspended in 500 μL of PBS 1X. Next, 300 μL of the re-suspended pool was subjected to DNAse treatment to eliminate background DNA with 20 U TURBO^™^ DNase (Ambion, Thermo Fisher Scientific, Waltham, MA, USA). Then, viral nucleic acids (NAs) were extracted with QIAmp Viral RNA Mini Kit (Qiagen, Inc., Valencia, CA), without carrier RNA, according to the manufacturer`s instructions. To enable the detection of both DNA and RNA viruses, total NAs were reverse-transcribed as previously described [23,24]. In short, SuperScript II (Life Technologies, California, USA) was used to retro-transcribe RNA to cDNA with primerA (5’-GTTTCCCAGTCACGATCNNNNNNNNN-3’). Second-strand cDNA and DNA were constructed with the primer sequences using Sequenase 2.0 (USB/Affymetrix, Cleveland, OH, USA). PCR amplification with AmpliTaqGold (Life Technologies, Austin, Texas, USA) was performed using primerB (5’-GTTTCCCAGTCACGATC-3’) with 30 cycles; this step was run in duplicate. The PCR products were purified and eluted in 15 μL using a Zymo DNA Clean and Concentrator kit (cat n° D4013, Zymo Research, USA) to yield enough DNA for the library preparation.

### Sequencing protocol

NGS sequencing was performed at SGB-UAB, Barcelona. dsDNA samples were quantified by Qubit 2.0 (Life technologies), and libraries were constructed using a Nextera XT DNA sample preparation kit (Illumina Inc). Samples were sequenced on Illumina MiSeq 2x300; all samples were multiplexed and distributed within three independent sequencing runs.

### NGS data processing

The quality of raw and clean read sequences was assessed using FASTX-Toolkit software, version 0.0.14 (Hannon Lab) [25]. The sequenced reads were cleaned by Trimmomatic version 0.32 [26] while the sequencing adaptors and linker contamination were removed. Low-quality ends were trimmed using a Phred score average threshold above Q15 over a running window of four nucleotides. Low-complexity sequences, mostly repetitive sequences that would affect the performance of downstream procedures in the computational protocol, were then discarded after estimating a linear model based on Trifonov's linguistic complexity and the sequence string-compression ratio. The discrimination criteria for that linear model assumes low complexity scores below the line having a -45° slope and crossing data distribution at 5% below the complexity inflexion point found by the model, which is specific to each sequence set. Finally, duplicated reads were removed in a subsequent step to speed up the downstream assembly.

### Sequence assembly and taxonomic assignment

Clean and filtered MiSeq reads were assembled using as parameters 90% identity over a minimum of 50% of the read total length in CLC Genomics Workbench 4.4 (CLC bio USA, Cambridge, MA) [27]. Afterwards, contigs longer than 100 bp were queried for sequence similarity using BLASTN and BLASTX (NCBI-BLAST [28]) against the NCBI complete viral genomes database [29,30], the viral division of the GenBank nucleotide database [31,32], and viral proteins from UniProt [33]. The species nomenclature and classification followed NCBI Taxonomy database standards and the basic Baltimore classification. The alignments reported by BLAST (High-scoring Segment Pairs, HSPs) were required to have an E-value lower than 10^−5^ and a minimum length of 100 bp to be considered for taxonomical assessment. On the basis of the best BLAST results and a 90% coverage cut-off, the sequences were classified into their most likely taxonomic groups of origin.

### Phylogenetic analysis

For *Anelloviridae*, phylogenetic trees were constructed based on the complete ORF1 region (with 75 reference sequences and a length alignment of 2551 bp), once contigs were properly aligned and trimmed. All the representative members of this family reported in humans were included as reference strains. Additionally, we also included some contigs longer than 1,500 bp that overlapped a large segment of ORF1 or a region upstream for individual trees. We compared each tree with the main tree generated from the reference strains to confirm equivalent distribution of species. In this manuscript, the following notation criteria were applied to name sequences on the phylogenetic trees: sequences covering ORF1, partially or not, were assigned to a number; contigs having some part outside ORF1 were identified with letters. For *Hepeviridae*, of the sequences mapped over the genome, we clipped the region that was present in all the sequence contigs under consideration. Then, the clipped region alignment was refined and some gaps were manually curated after visual inspection to improve the resulting alignment score. A reference phylogenetic tree was calculated from an alignment of 7483 bp with 22 known complete genomic sequences (19 of the genotype 3) as previously described [34]. Partial contig sequences aligning to a given particular region produced an equivalent tree. Those sequences were manually placed in the main tree according to the corresponding branches position on the equivalent trees, yet they are shown on the main reference tree as numbers or letters next to reference sequence identifier. All the alignments were produced by Geneious 10^®^ as well as the phylogenetic trees, which were computed using the neighbour-joining method under the Jukes Cantor model. The robustness of the trees was assessed by bootstrap analysis of 1000 replicates each; finally, the branches are proportional to the corresponding phylogenetic distance.

### Ethical statement

The study has been approved by the corresponding ethical committee: ethical committee on clinical investigation and research projects of the Hospital Universitari Vall D'Hebron (N° 185; date: 4/2/2011). Serum samples were pooled at the hospital and for this study we do not have information on the identity of the patients.

## Results

**Fig 1:**
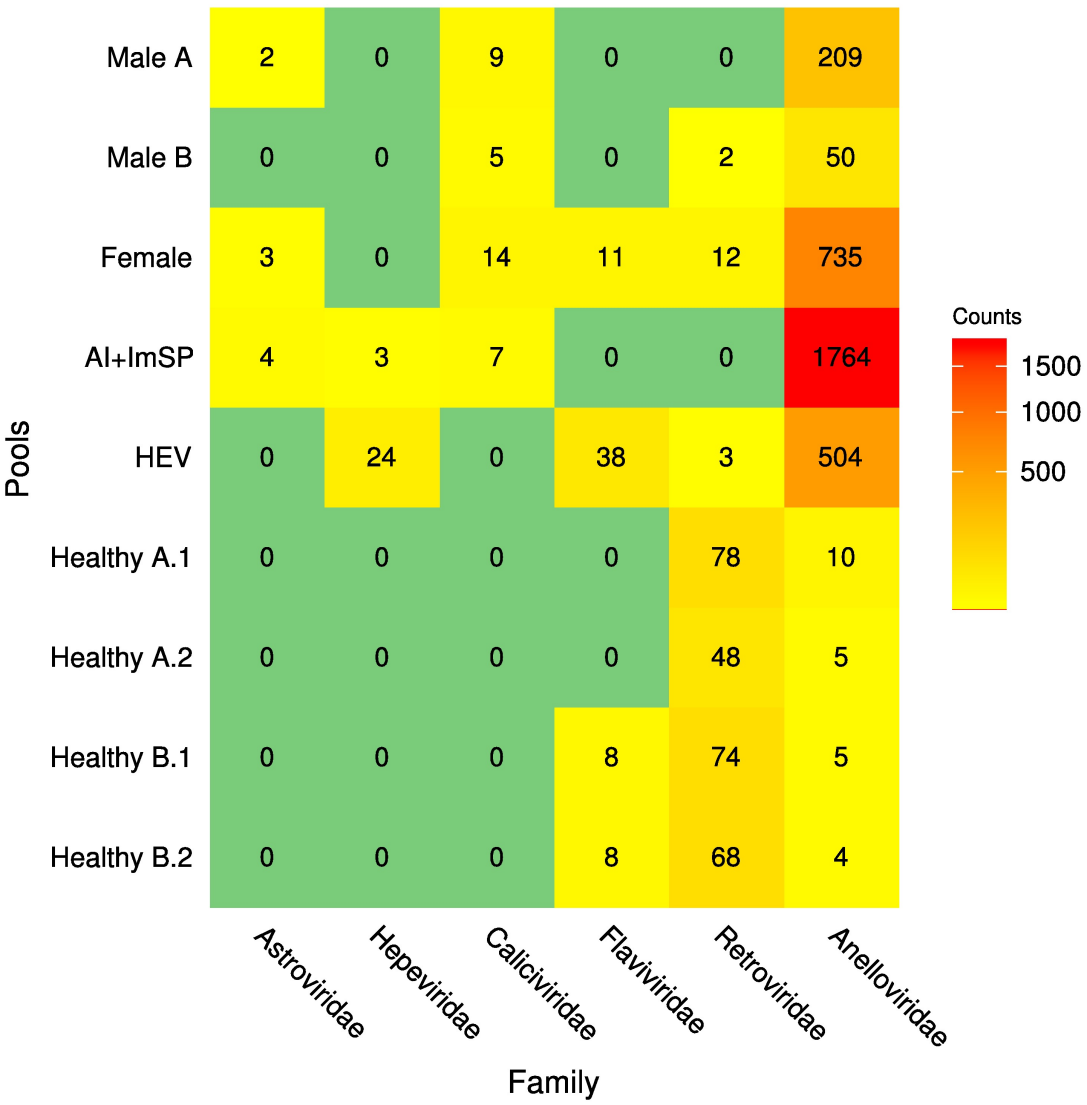
Heatmap describing number of contigs identified in each pool after their characterization and classification into taxonomic groups. Rows correspond to pooled samples whilst columns to families mapped at least to one sample. Numbers within each cell represent the number of sequences that had at least a positive BLAST hit to into known species and passed all the selection criteria. The colours range from yellow to red (low to high abundance respectively); green means that sequences were not detected for that group.

Nine libraries, consisting of 62 serum samples (32 of patients with unknown hepatitis, 10 of known HEV infections and 20 heathy volunteers), were obtained and sequenced using paired-end 300-base runs on the Illumina MiSeq platform, generating a total of 48 million reads (see Table 1 for a summary of the sequencing statistics for individual pools). Raw reads were binned by pool-based library barcodes and quality-filtered, leaving 30.5 million high-quality reads, which were assembled *de novo* within each pool subset. The resulting sequence contigs and singletons were compared to NCBI complete viral genomes, the viral division of the GenBank nucleotide database, and viral proteins retrieved from UniProt. Most of the viral sequences detected were related to the *Anelloviridae*, *Astroviridae*, *Caliciviridae*, *Hepeviridae*, *Flaviviridae* and *Retroviridae* families (Fig 1); those near-to-complete or partial genomes were characterized and are described in the following sections.

**Table 1:** Summary of the sequences produced for each pool of serum samples in the sequencing experiment. All read counts correspond to total values, and the paired-reads real counts are half the values shown in the table. PE: paired-end reads; SE: single-end reads.

Pool ID	Number of samples	Raw Reads (PE MiSeq)	Clean Reads (PE + SE)	Contigs (after assembly)
Male A	8	5,255,854	3,614,220 + 6,928	43,188
Male B	8	2,669,124	1,769,992 + 4,738	19,000
Female	8	12,029,238	7,470,502 +18,074	83,518
Ai+ImSP	8	6,000,606	3,887,728 + 197,320	5,889
HEV	10	8,145,076	5,769,136 + 2,025	13,060
Healthy A.1	10	3,413,928	1,873,370 + 286	189,820
Healthy A.2		3,588,692	1,796,830 +298	166,167
Healthy B.1	10	3,457,150	2,119,224 +250	227,469
Healthy B.2		3,494,586	1,934,704 + 4	185,359

Volunteer samples that were analysed in the Healthy pools, in duplicate, show similar number of reads, and contigs. Additionally, the same families were found in those replicates, demonstrating that those results are highly consistent across samples (Table 1 and Fig 1).

### Hepeviridae

**Fig 2:**
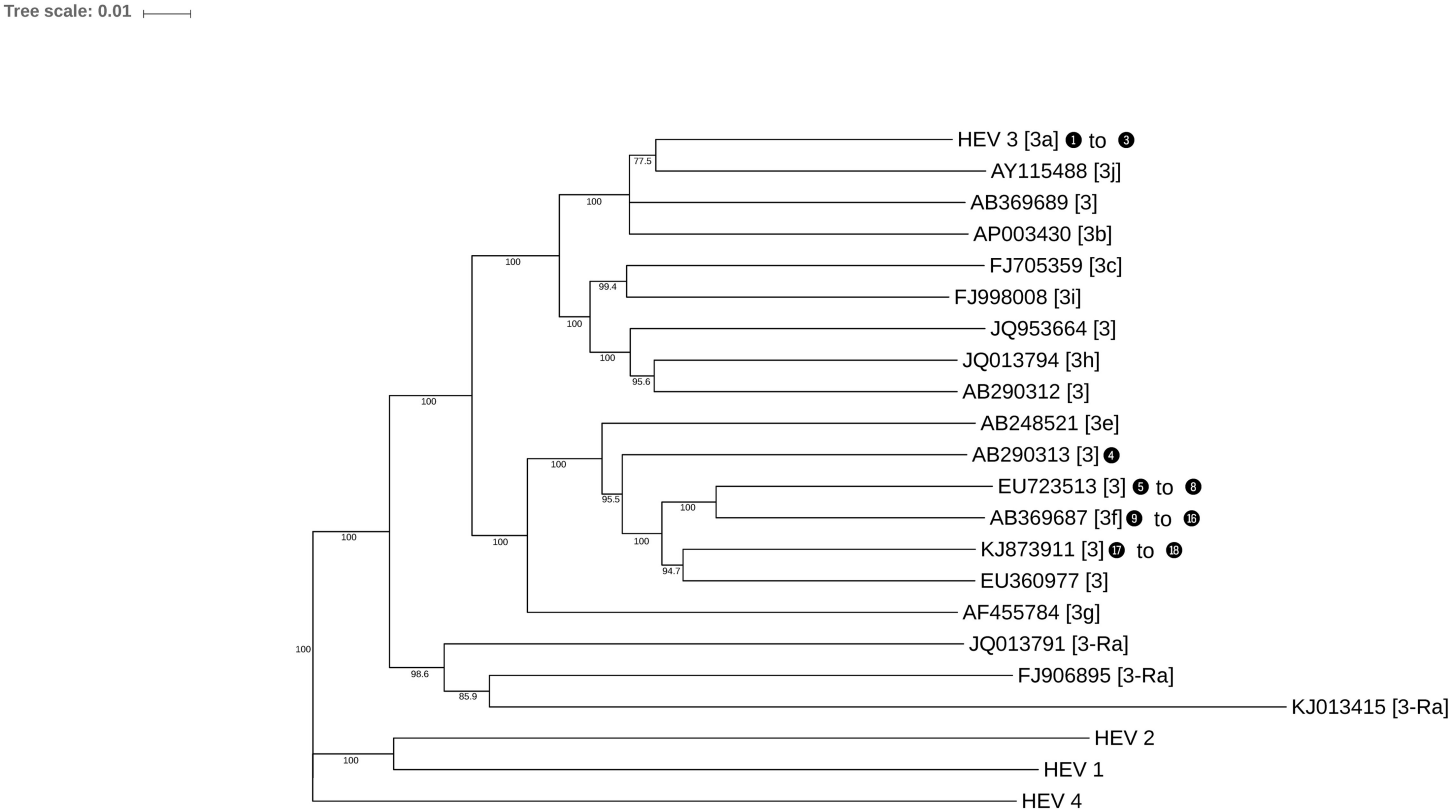
Phylogenetic tree of *Hepeviridae* based on complete genomes, including the main members of genotype 3. Numbers in blank bullets correspond to contigs identified in the HEV and Ai+ImSP pools (see Table 2); they are located beside the reference sequence where specific individual alignments of sequenced fragments over the same region in the reference sequences generated an equivalent tree topology (further results available from S1 Supporting Information). Labels within the square brackets define the species subtype. Small numbers on the tree branches show the bootstrap score of those branches.

A total of 27 contigs were matched to sequences of the *Hepeviridae* family. The HEV and Ai+ImSP pools produced sequences related to this family. A total of 76.1% (5,508 of 7,238 bp) of the HEV genome was sequenced from the HEV pool, with an average pairwise identity of 85.5% against the genotype 3 HEV (AF082843, Reference sequence genotype 3 ICTV). To identify the genotypes present in the pools and because metagenomics amplified different regions of the genome at random, individual phylogenetic trees were computed from contigs mapping over the same reference genome locations. The individual trees were compared to a reference species tree based on the reference-genomic sequences. Contigs that produced trees similar to the reference are marked in Fig 2 using numeric indexes, and information about each of those contigs is displayed on Table 2. On this table each contig is identified by its name (Contig ID), the contig length, its alignment identity percent to the homologous sequence from the blast HSPs, and confidence bootstrap value of the branch where it is placed on the corresponding phylogenetic tree. We were able to generate phylogenetic trees similar to the reference for eighteen contigs (the individual trees are available in S1 Supporting Information). Fifteen contigs from the HEV pool aligned to genotype 3f or closely related genotypes. The three contigs from the Ai+ImSP pool aligned with genotype 3a.

**Table 2:** Summary of similarity searches for those detected from the HEV and Ai+ImSP pools. The first column corresponds to the numbers in the black bullets shown on some of the branches of the *Hepeviridae* phylogenetic tree from Fig 2.

Code	Pool	Contig ID	Length	%Identity	Bootstrap
1	AI+IMSP	contig_953	992	91.63	100.0
2	AI+IMSP	contig_1893	686	91.40	97.4
3	AI+IMSP	contig_3606	412	91.02	84.1
4	HEV	contig 3810	573	86.83	60.0
5	HEV	contig_2453	1,416	91.05	68.4
6	HEV	contig_533	590	90.51	70.0
7	HEV	contig_1542	575	89.04	76.7
8	HEV	contig 749	541	88.54	51.3
9	HEV	contig_6571	1,572	91.35	99.9
10	HEV	contig_747	1,415	88.30	74.6
11	HEV	contig _3424	1,032	90.31	83.3
12	HEV	contig_6979	944	91.58	100.0
13	HEV	contig_7146	929	89.26	95.0
14	HEV	contig_8370	557	93.33	96.0
15	HEV	contig 10460	314	86.29	95.4
16	HEV	contig 3914	297	87.21	65.2
17	HEV	contig_1444	1,534	87.11	98.3
18	HEV	contig 3007	333	89.33	86.7

### Anelloviridae

**Fig 3:**
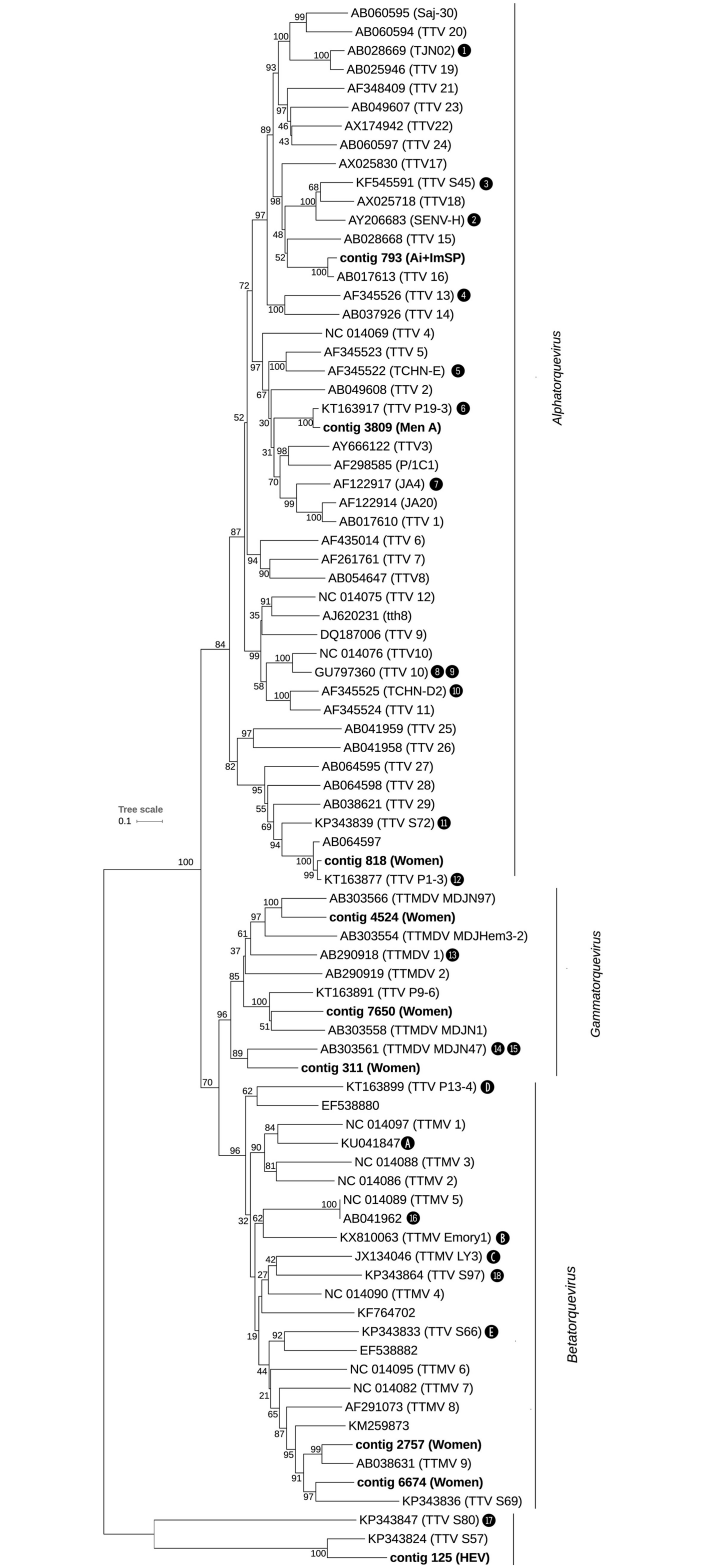
Phylogenetic tree for the *Anelloviridae* family based on ORF1 region and including only contigs that fully overlap with that region. Numbers and letters within black bullets refer to contigs longer than 1,500 bp (see Table 3) that partially aligned with ORF1 or with the ORF1 upstream region, respectively. See Fig 2 for further details about notation used in this tree.

A total of 3,286 contigs matched sequences from the *Anelloviridae* family. All the pools produced sequences related to this family; however, the number of contigs was significantly higher in the pools with signs of hepatitis compared to the healthy pools (Wilcoxon rank-sum test, *p* = 0.009) and much more abundant in the Ai+ImSP pool (Fig 1). Contigs completely covering the ORF1 region of *Anelloviridae* family—or longer than 1,500 bp and overlapping this region—were found in the male A (less than 48 years old), female, HEV, and Ai+ImSP pools. Those particularly long sequences were used to build a phylogenetic tree to obtain a more accurate characterization of the species (Fig 3 and Table 3). The main members detected were Torque Teno Viruses (TTV—genus *Alphatorquevirus*) 1, 5, 10, 11, 13, 16, 18, 19, SEN virus H, Torque Teno Mini Viruses (TTMV—genus *Betatorquevirus*) 5, 9 and 18, Torque Teno Midi Viruses (TTMDV—genus *Gammatorquevirus*) 1, MDJN47, MDJN97, and other unclassified anelloviruses: TTV P19-3 (KT163917), TTV S72 (KP343839), TTV P1-3 (KT163877), TTV P13-4 (KT163899), TTMV Emory1 (KX810063), TTV S97 (KP343864), TTMV LY3 (JX134046), TTV S66 (KP343833), TTV S69 (KP343836), TTV S45 (KF545591), TTV P9-6 (KT163891), TTV S80 (KP343847), and TTV S57 (KP343824). Furthermore, contigs matching to the last two reference sequences do not belong to the three known genera of *Anelloviridae* previously identified in humans; thus, it seems they define a new cluster/genus for this family. Moreover, 60% (19/32) of the longest contigs have less than 80% identity to the already described sequences from the NCBI database. Table 3 shows the contigs that were considered for this phylogenetic analysis; each contig is identified by its name (contig ID), sequence length in bp, alignment identity percent to the homologus sequence from the BLAST HSPs, and confidence bootstrap value of the branch where it is placed on the corresponding phylogenetic tree (individual trees are provided in the S2 Supporting Information). Fewer and shorter contigs were found in the pools from healthy individuals in comparison with the other pools (median of 300 bp); they correspond to TTV 1, 19 and TTMV 6.

**Table 3:** Summary information for contigs longer than 1,500 bp that were found in the pooled samples and assigned to the *Anelloviridae* family. The number and letter codes from the first column (Code) correspond to those in the blank bullets shown on some of the branches of the phylogenetic tree from Fig 3. Those without codes were placed directly on the tree, as they defined new branches.

Code	Sample	Contig ID	Length (bp)	Sequence Name	% Identity	Bootstrap
	Male A	contig_3809	2,798	TTV P19-3	92.8	100.0
D	Male A	contig_1199	1,512	TTV P13-4	69.3	68.0
10	Male A	contig_7929	1,875	TCHN-D2/TTV 11	79.2	100.0
12	Female	contig_1	1,548	TTV P1-3	92.4	100.0
11	Female	contig_129	1,506	TTV S72	79.8	100.0
14	Female	contig_16376	1,513	TTMDV MDJN47	68.3	91.9
	Female	contig_2757	2,142	TTMV 9	76.6	99.0
	Female	contig_4524	1,977	TTMDV MDJN97	72.1	100.0
1	Female	contig_5911	1,500	TJN2/TTV19	88.9	100.0
A	Female	contig_626	1,503	TTMV 18	89.7	100.0
	Female	contig_6674	2,381	TTV S69	71.2	97.0
	Female	contig_818	2,264	TTV P1-3	95.1	99.0
7	Female	contig_9035	2,243	JA4	94.0	100.0
4	Female	contig_1475	1,899	TCHN-A	87.5	100.0
13	Female	contig_1946	1,644	TTMDV1	71.6	100.0
2	Female	contig_268	1,951	SENV-H	90.1	100.0
	Female	contig_311	2,086	TTMDV MDJN47	66.6	89.0
8	Female	contig_6533	1,530	TTV10	83.3	100.0
	Female	contig_7650	1,730	TTV P9-6	69.6	51.0
17	AI+IMSP	contig_1199	1,586	TTV S80	66.8	99.8
	AI+IMSP	contig_793	2,367	TTV 16	90.8	100.0
15	AI+IMSP	contig_1013	1,687	TTMDV MDJN47	66.0	95.8
9	AI+IMSP	contig_1709	1,832	TTV 10	85.7	100.0
18	AI+IMSP	contig_2151	1,985	TTV S97	65.6	79.0
B	HEV	contig_118	1,781	TTMV Emory1	68.6	68.0
3	HEV	contig_2837	1,845	TTV S45	86.6	100.0
	HEV	contig_125	2,303	TTV S57	70.1	100.0
C	HEV	contig_2	1,923	TTMV LY3	66.6	79.0
5	HEV	contig_236	1,643	TTV TCHN-E	72.0	99.9
6	HEV	contig_2366	1,514	TTV P19-3	93.8	100.0
16	HEV	contig_506	2,046	TTMV 5	73.7	96.0
E	HEV	contig_66	1,904	TTV S66	71.9	49.0

### Caliciviridae

A total of 35 contigs between 200 and 654 bp aligned to the *Caliciviridae* family. They were found in the male A and B, female and Ai+ImSP pools. No sequences of this family were detected in healthy volunteer pools. All contigs were assigned to sapovirus Hu/Nagoya/NGY (AB775659), genogroup 5 strain 2 (GV.2), with identities varying between 97% and 100%. Those contigs map over several regions of the non-structural protein and major structural protein, including eleven that aligned to a partial capsid fragment.

### Astroviridae

As few as eight contigs between 214 and 493 bp long matched the *Astroviridae* family. They were found in the male A (less than 48 years old), female, and Ai+ImSP pools. No sequences of this family were detected in the healthy-volunteers pools. These contigs correspond to a recently discovered astrovirus, clade VA strain 3 (VA3, also known as HMO-C) (7 matching JX857868, 1 matching JX083288), with identities ranging from 97% to 100%.

### Flaviviridae

A total of 65 contigs between 219 and 2778 bp matched the *Flaviviridae* family. They were found in the female, Ai+ImSP, and healthy B1 and B2 pools. All the sequences aligned to several entries of GB virus C from GenBank, with identities between 97% and 100%.

### Retroviridae

In this case, 285 contigs between 300 and 1,032 bp were assigned to the *Retroviridae* family. They were found in the male B (more than 48 years old), female, and HEV pools and in all healthy pools. All the sequences matched several entries of human endogenous retrovirus type K and HCML-ARV with identities greater than 70%.

The raw sequencing data used to perform this analysis along with the FASTQ file are located in the NCBI Sequence Read Archive; BioProject (PRJNA379441).

## Discussion

The aim of this study was to investigate viruses infecting patients diagnosed with acute hepatitis. Different groups of patients presenting with acute hepatitis but without serological infection markers of the most common viral hepatitis were studied to determine possible causal agents of non-A-E hepatitis. Our findings demonstrate the presence of a high variety of viral sequences in pools of patients with hepatitis of unknown aetiology.

HEV viruses were detected in two pools (HEV and Ai+ImSP). We found a variety of contigs related to genotype 3f in HEV pools. Genotype 3f has been described in hepatitis outbreaks in Catalonia [35], Spain [36] and the south of France [37]. This strain has also been related to swine and wild boar consumption, which can be considered a food-borne and an emerging zoonotic infection [35,38,39]. Individual samples from the Ai+ImSP pool were re-analysed afterwards by nPCR, and one patient was identified as HEV-positive in this second round, which would explain the presence of HEV contigs in this pool. Metagenomics approaches have the advantage of identifying more than one genotype in the pools; this facilitates description of traces of possible multiple infections in a single sample.

We have found at least three different kinds of *Anelloviridae* contigs: a) contigs that match previously characterized sequences; b) contigs that are closely related to unclassified sequences; and, c) contigs poorly related to classified and unclassified sequences (potential new viruses). The demarcation criteria of the genus establish a cut-off value of 35% nucleic-acid identity in the ORF1 region. Due to the number of quasispecies discovered in this family [40], it is difficult to establish a clear cut-off at the species level.

We also describe in this paper viruses that have been previously associated with hepatitis such as TTV-1, 11, 16 and SEN virus H [14,41]; other viruses have been recently described in serum samples from HIV patients (P19-3, P13-4, P9-6, P1-3); yet other sets were described in patients with various conditions, including lymphocytic leukaemia (TTV 10) [42], gingival periodontitis (TTMV 18) [43], haemophilia (TTMDV MDJN47 and MDJN97) [44] and in pregnant women whose offspring developed leukaemia and lymphomas (TTV S45, S57, S66, S69, S72, S80 and S97) [45].

Metagenomics analyses are driving the discovery of new potential sequences in this family; Bzhalava et al. (2016) described for first time a group of sequences detected from human samples, spawning a new branch of the *Anelloviridae* family. We found two contigs (125 and 1199) falling into this new potential genus of *Anelloviridae*, yet they have less than 70% of identity to those sequences, which were described in serum samples from pregnant women. Such results suggest that there will be more viruses within this family that have not yet been identified.

TTV-1, the first member identified in the *Anelloviridae* family, was reported in hepatitis patients in whom no causative agents were detected [12]. This family includes three genera that have been identified in humans: *Alphatorquevirus* (TTV), *Betatorquevirus* (TTMV), and *Gammatorquevirus* (TTMDV) [46]. However, the role of those viruses in hepatitis or in other diseases remains uncertain [14,40,47]. Numerous recent studies have demonstrated a prevalence between 5 and 90% in the blood of the general population, depending on the geographic region [40]. Moreover, the genetic diversity among anelloviruses is far greater than it is within any other group of ssDNA viruses. The considerable genetic heterogeneity is exemplified by the large number of highly divergent sequences being identified in this family. There are at least 41 species infecting humans that are recognized by the ICTV based on the ORF1 region [46]. Some viruses, such as TTV 1, 12, 13, 16, SEN virus D and H, have been considered potential causal agents of hepatitis [14,48–50].

Unfortunately, anelloviruses cannot be propagated *in vitro* due to the lack of compatible cell systems. However, they have a high *in vivo* replication capacity. Infection with TTV is characterized by persistent lifelong viremia in humans, with circulation levels of up to 10^6^ genomic copies/ml in the general population [14,40]. TTV replicates in the liver and is excreted at high levels in bile and faeces [51]. Additionally, other studies have shown that this virus does not have a particular tropism [40,52]. Metagenomic analyses have also shown that TTV is a common finding in several sample types [53]. For that reason, determining the causative factors of illness can be difficult.

An increased number of contigs aligning to anelloviruses was observed in this study, however, these findings not necessarily may support the hypothesis that these viruses are the causative agents. Previous studies have suggested titres of TTV in plasma as an indicator of immune status [54]. Another study showed that anellovirus load in plasma increases substantially during immunosuppressive therapy and in immunocompromised patients [55]. Shotgun sequencing from plasma samples that were collected over several months post-transplantation also revealed that viral loads increased, whereas the bacterial composition remained unchanged [56].

The results described in this study also show the presence of sapovirus strain GV.2 in all the pools of patients with clinical hepatitis of unknown aetiology. This strain has been recently characterized from faecal samples from a suspected foodborne gastroenteritis outbreak in Japan using a metagenomics sequencing approach [57]. Partial fragments of that virus were described early from another gastroenteritis outbreak in Italy [58], in river water from Barcelona (the same region where this study was conducted) [59], and in wastewater from Japan [60], suggesting prevalent circulation of this virus around the world. Sapovirus are positive-sense single-stranded RNA viruses from the family *Caliciviridae*. Members of this family are known to cause gastroenteritis with self-limited infections and low mortality rates; severe infections or serious clinical complications are usually reported in immunocompromised patients [61]. Further research would be required to analyse the possible pathogenic role of sapovirus GV.2 in our study.

Few contigs of the *Astroviridae* family were detected in this work. Astrovirus VA3 was identified in most of the pools of hepatitis of unknown aetiology. However, those contigs were less abundant and shorter than the sapovirus contigs. The first description of astrovirus VA3 was from the stool of paediatric patients with diarrhoea from India [62], and it was later completely sequenced [63]. This virus has also been described in stools from southern China [64], Kenya, and the Gambia [65]. However, the role of this virus in health and disease remain largely unknown.

The potential pathogenic role of sapovirus GV.2 and astrovirus VA3 in blood remains still uncertain. Although astroviruses and sapoviruses are considered gastrointestinal pathogens, viral RNA and infectious particles have been recovered from extraintestinal organs in both animals and humans. Examples in animals implicate astroviruses as the cause of hepatitis in ducks [66] and the isolation of murine astroviruses in mouse liver [67]. With respect to sapovirus less information is available; an isolation of sapovirus in a liver of a spotted hyena [68]. Our results suggest that the presence of these viruses in pools from patients with non A-to-E hepatitis, including the AI+ImSp pool, merits further research, since there is no previous evidence relating those viruses to hepatitis.

GB virus C, also known as pegivirus or hepatitis G virus, is a human virus of the *Flaviviridae* family that is structurally and epidemiologically closest to hepatitis C virus [13]. Most GBV-C infections appear to be asymptomatic, transient, and self-limiting, with slight or no elevation of ALT levels. Those infections are rarely identified and very difficult to evaluate. The role of GBV-C in the aetiology of hepatitis has not been fully established [69]. Moreover, it is commonly reported in metagenomics studies [53], suggesting its limited role in the development of illness, including hepatitis. We have detected this virus in one healthy pool and in a hepatitis pool; our results support the hypothesis that this species may be widely distributed within the population.

Human endogenous retroviruses (HERVs) are remnants of germ-line retrovirus integration and are considered functionally defective [70]. They have been described in metagenomics studies at high levels [55,70] without association with any particular pathology [71]. Our findings support previous results pointing out that this virus is present in healthy people.

It is important to recognize that the use of serum samples to describe the virome may have some minor limitations as a decreased sensitivity to detect integrated proviruses (e.g. HIV-1), episomal viruses (e.g. herpesviruses) [72]. Furthermore, giant viruses may also be under-represented due to the filtration process [73]. However, serum samples predominantly contain host DNA which can also affect the sensitivity of viral detection [74]; if host and viral NA cannot be easily separated, the resulting fraction of viral sequences relative to the host DNA would be extremely low [53]. Pretreatments protocols for viral enrichment have to be taken into consideration in order to get a better approximation to the whole virome and the interaction between virus population in future studies.

## Conclusions

In summary, metagenomics was applied in this study to detect a broad spectrum of viral species based on sequences found in pooled samples, including HEV in pools of patients with confirmed HEV; these samples allowed the characterization of the most prevalent genotypes. Additionally, we were able to identify a diverse population of anelloviruses, including novel undescribed sequences, in patients with acute hepatitis of unknown aetiology. Furthermore, sapovirus GV.2 and astrovirus VA3, viruses recently reported as cause of gastroenteritis, were also found exclusively in those pools. We did not attempt to determine causality or to describe epidemiologic results; our purpose was to characterize the virome of patients diagnosed with hepatitis to describe new potential causal agents. The role of these viruses as possible causal agents of hepatitis of unknown aetiology remains open to further studies. Finally, reproducibility between replicates in the pools of healthy volunteers supports the consideration of the metagenomics as a robust detection method for viral species. Metagenomics analyses offer unprecedented possibilities for diagnostics, characterization and identification of possible co-infections of rare and novel viruses that will be relevant to understanding the aetiology of current pathologies without known causative agents.

## Supporting information

S1 Supporting Information Individual phylogenetic trees computed from contigs over reference genome locations in HEV. (DOCX)

S2 Supporting Information Individual phylogenetic trees computed from contigs over reference genome locations in *Anelloviridae* family. (DOCX)
