# Supplementary material for: Identification of sapovirus GV.2, astrovirus VA3 and novel anelloviruses in serum from patients with acute hepatitis of unknown aetiology
Source: PLoS One. 2017 Oct 5;12(10):e0185911. doi: 10.1371/journal.pone.0185911 (PMC5628893; doi:10.1371/journal.pone.0185911)
Supplement: S1 Supporting Information — (DOCX) [file pone.0185911.s001.docx]

**S1 Supporting Information: Individual phylogenetic trees computed from contigs over reference genome locations in HEV.**

**HEV SH5**

**Contig_953**

**
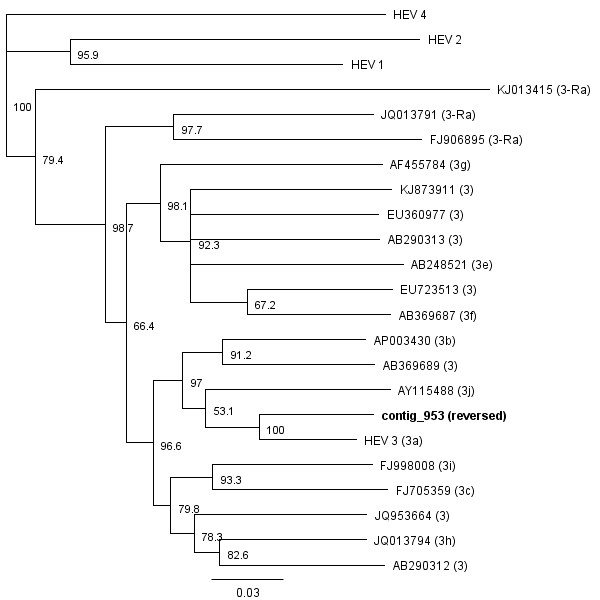
**

**Contig 3606**

**
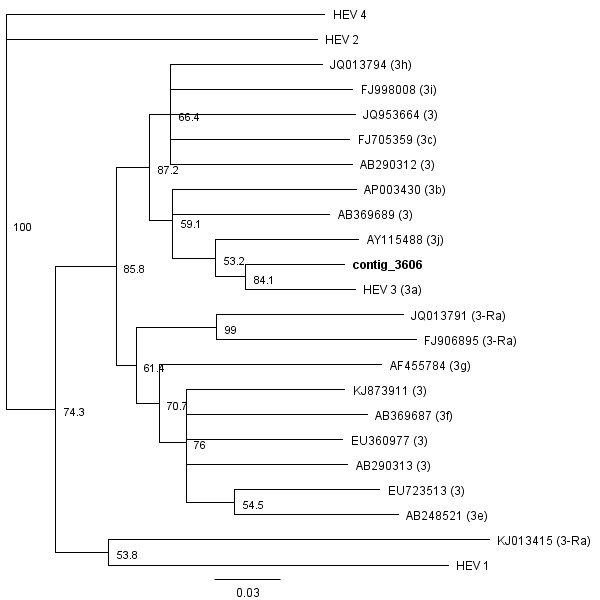
**

**Contig 1893**

**
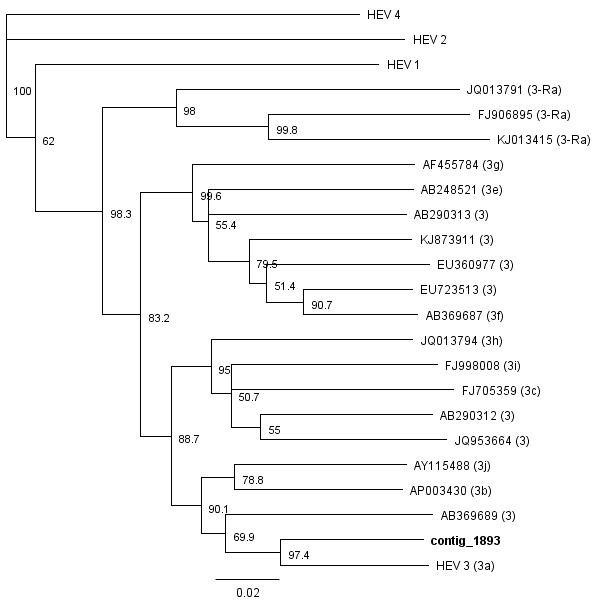
**

**HEV SH6**

**Contig 533**

**
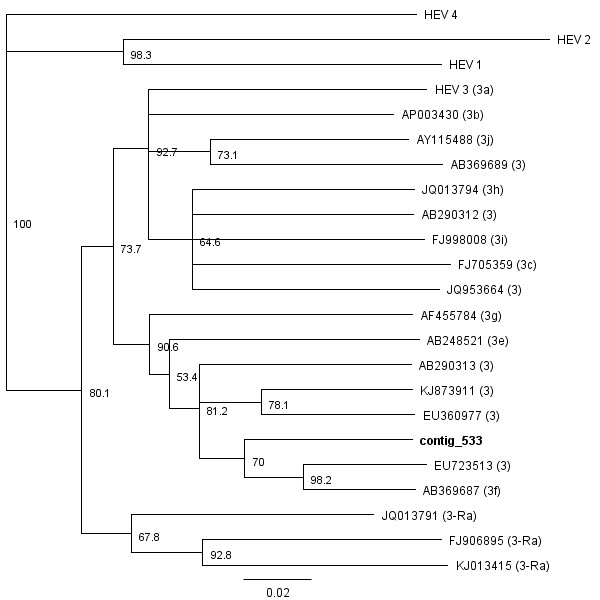
**

**Contig 747**

**
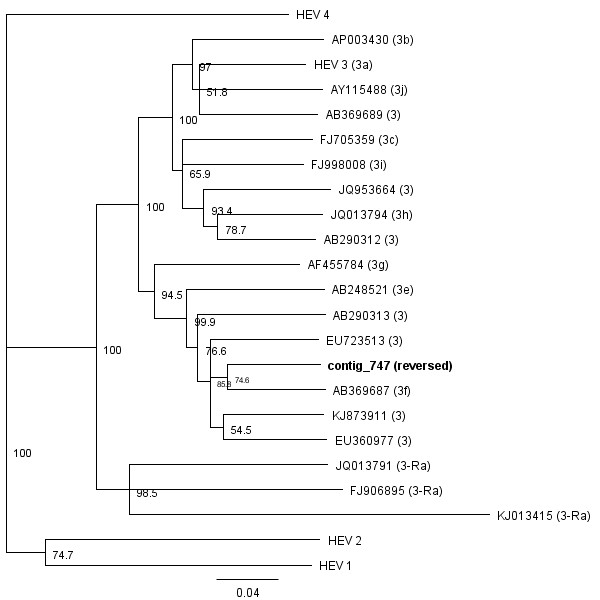
**

**Contig 749**

**
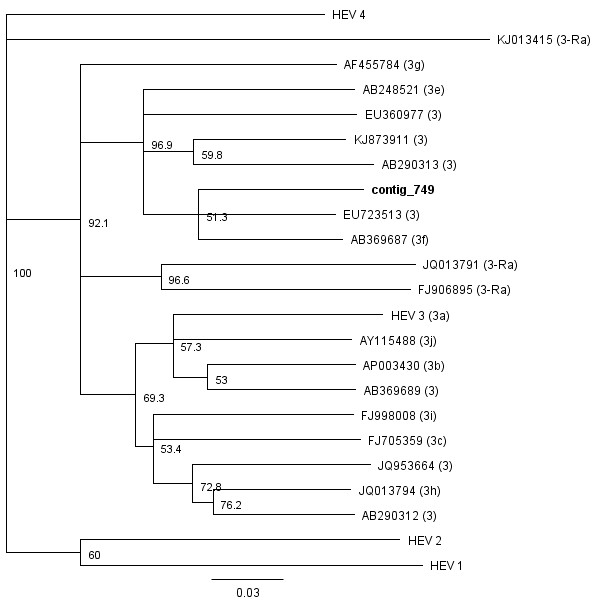
**

**Contig 1444**

**
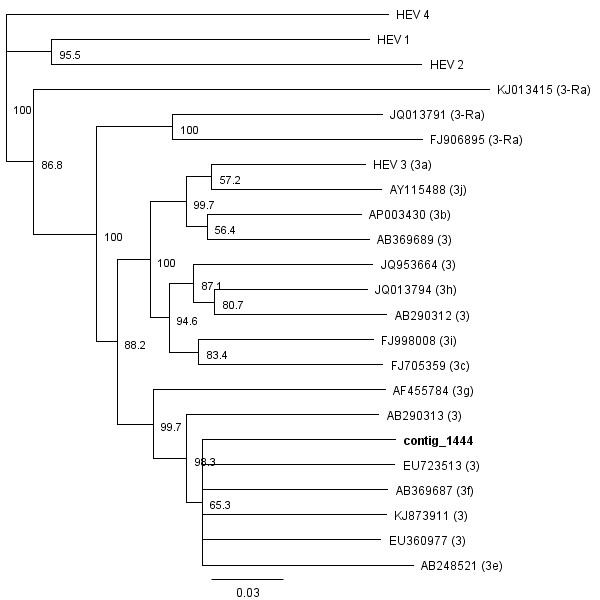
**

**Contig 1542**

**
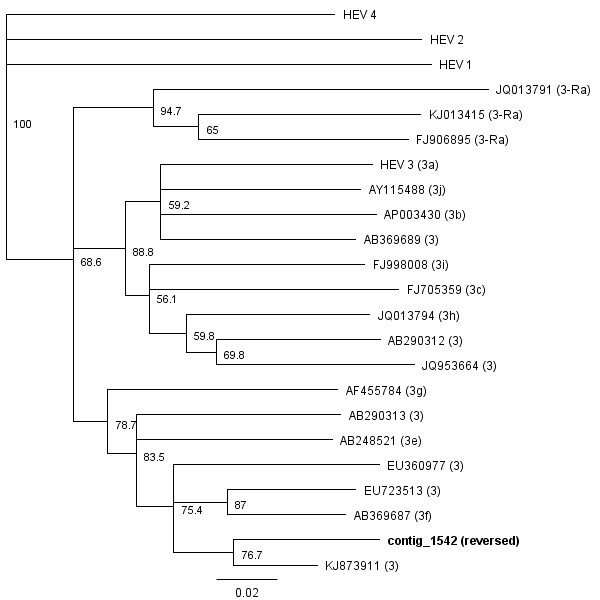
**

**Contig 2453**

**
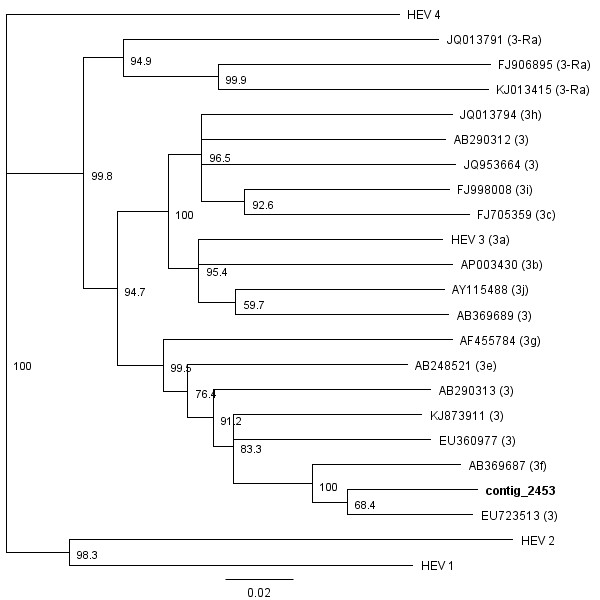
**

**Contig 3007**

**
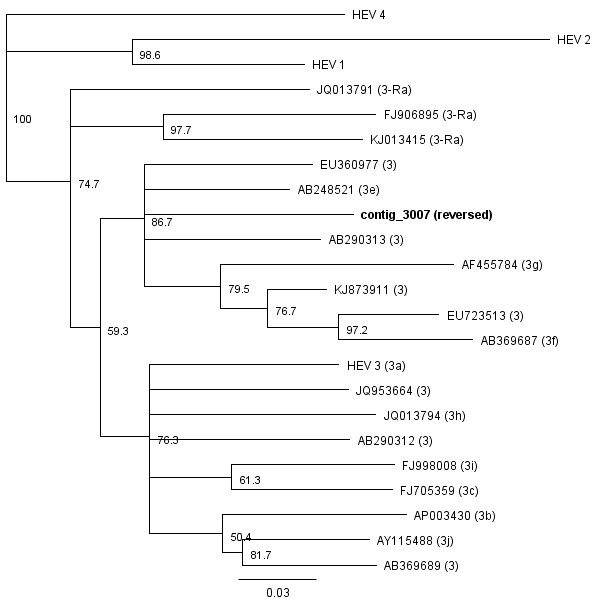
**

**Contig 3424**

**
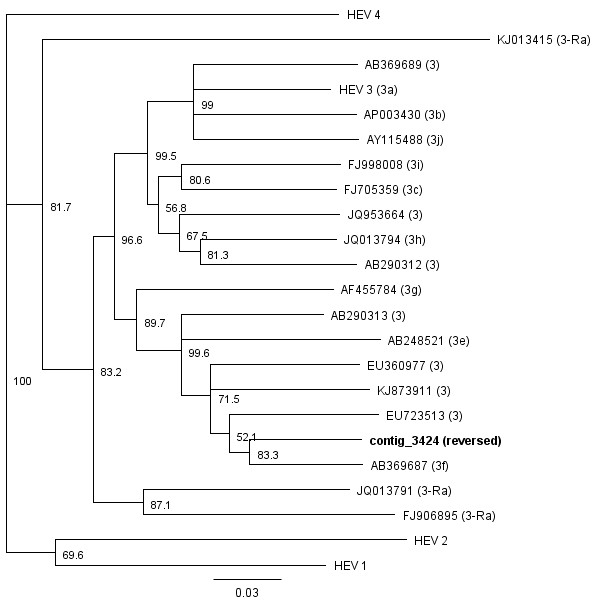
**

**Contig 3810**

**
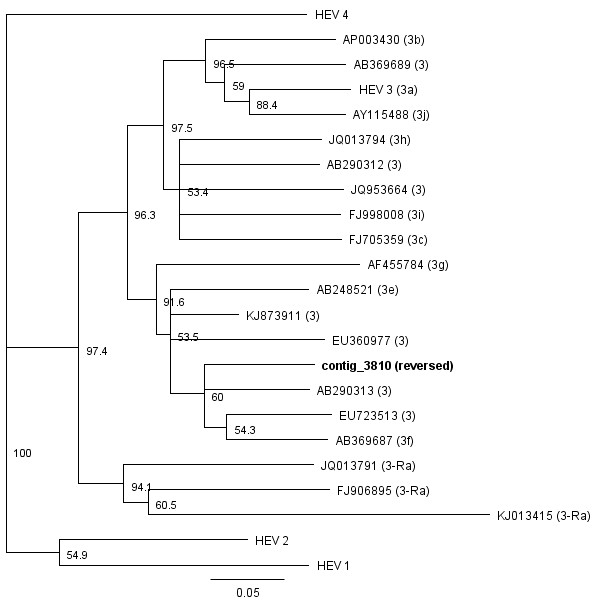
**

**Contig 3914**

**
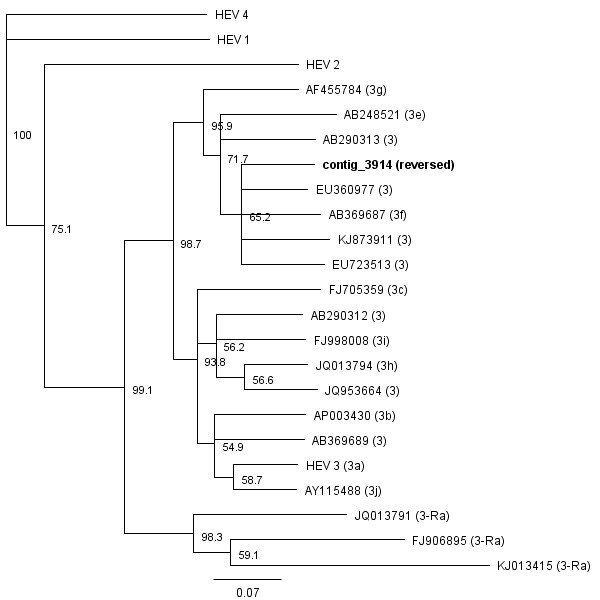
**

**Contig 6571**

**
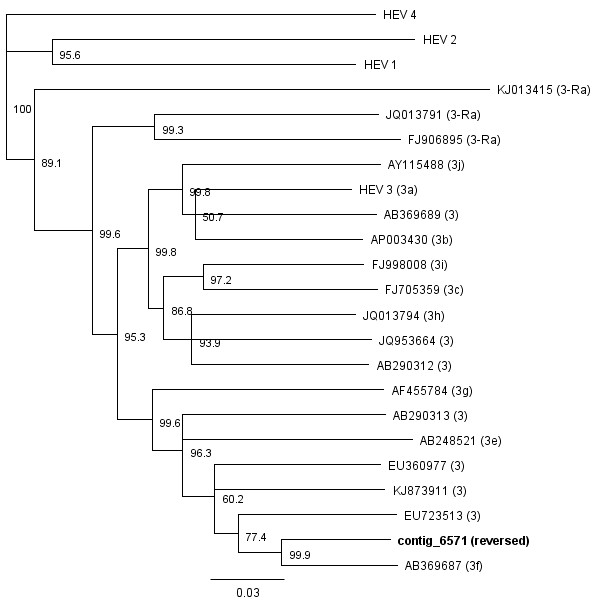
**

**Contig 6979**

**
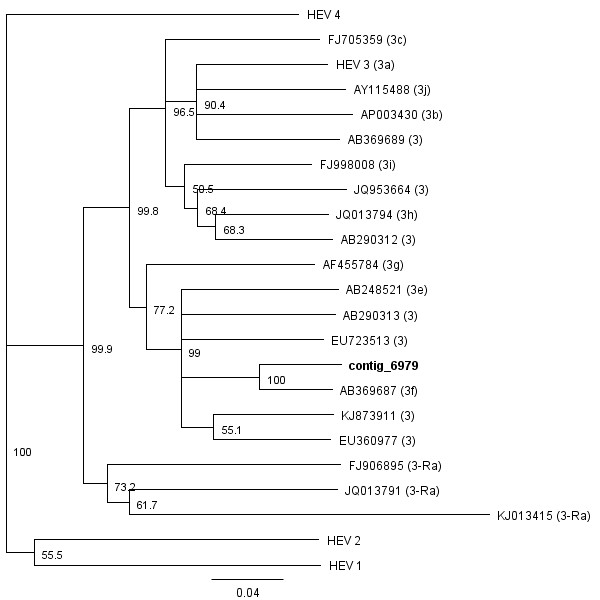
**

**Contig 7146**

**
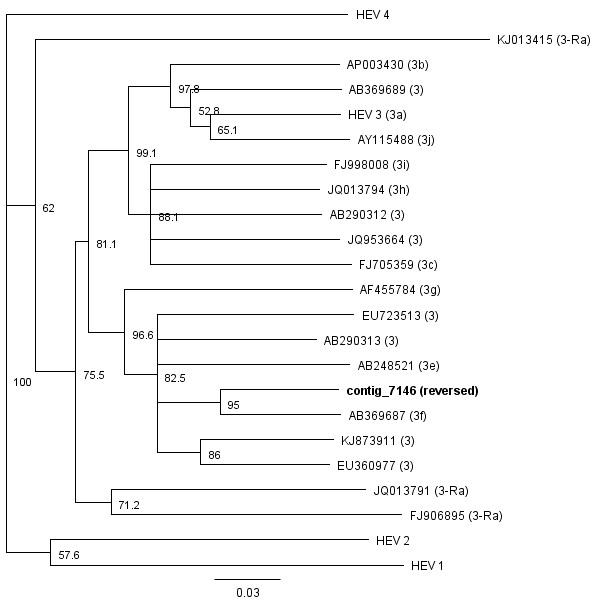
**

**Contig 8370**

**
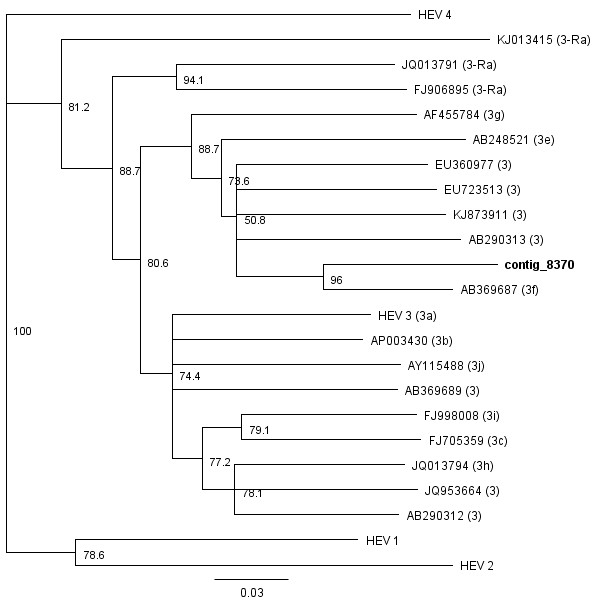
**

**Contig 10460**

**
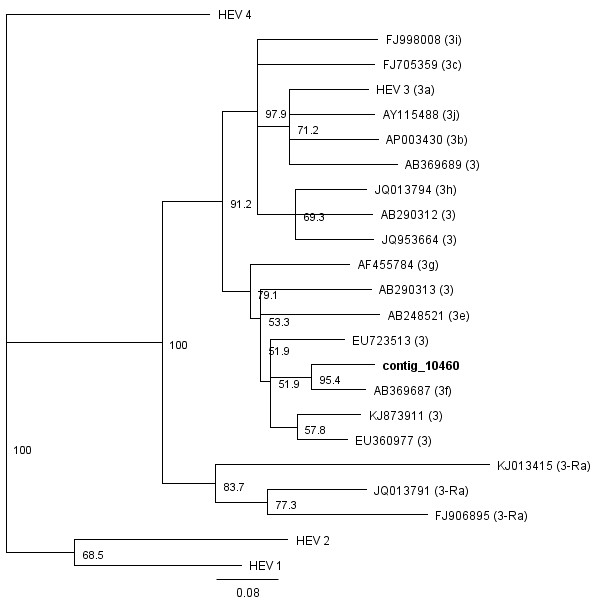
**
