# Supplementary material for: Identification of sapovirus GV.2, astrovirus VA3 and novel anelloviruses in serum from patients with acute hepatitis of unknown aetiology
Source: PLoS One. 2017 Oct 5;12(10):e0185911. doi: 10.1371/journal.pone.0185911 (PMC5628893; doi:10.1371/journal.pone.0185911)
Supplement: S2 Supporting Information — (DOCX) [file pone.0185911.s002.docx]

**S2 Supporting Information: Individual phylogenetic trees computed from contigs over reference genome locations in *Anelloviridae* family.**

**TTV**

**Contig 1**

**
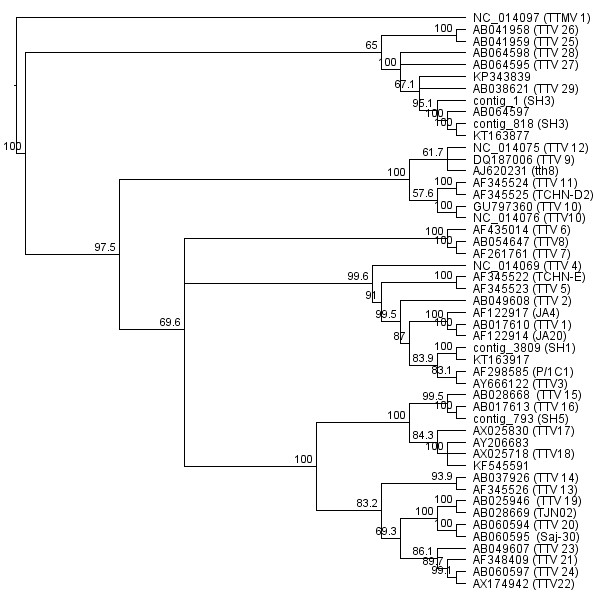
**

**Contig 129**

**
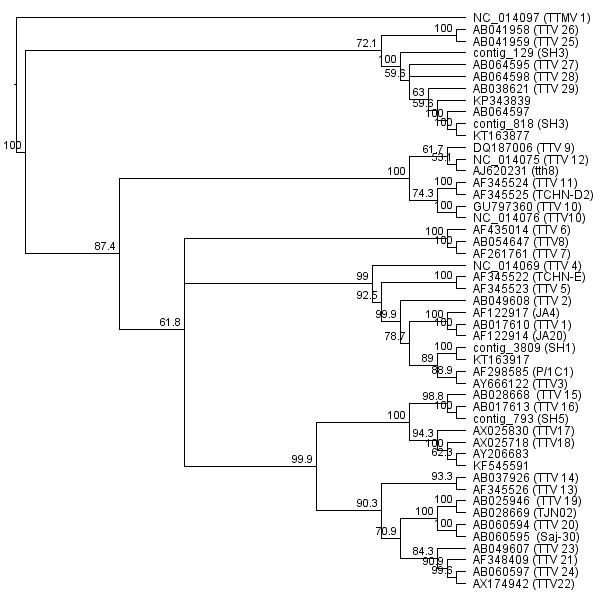
**

**Contig 236**

**
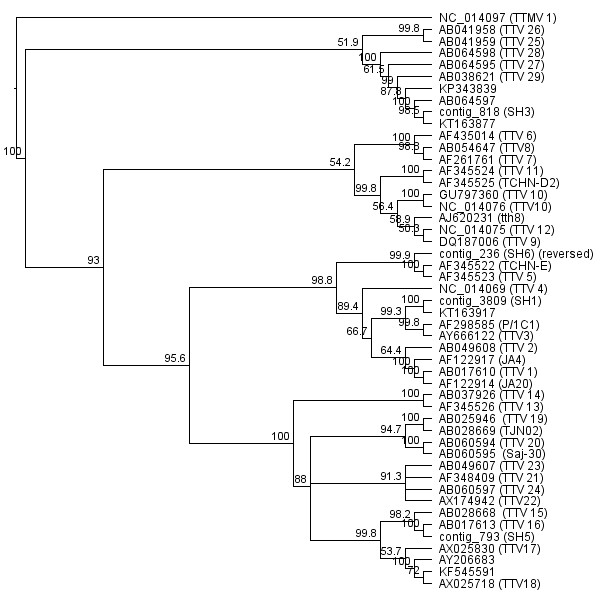
**

**Contig 268**

**
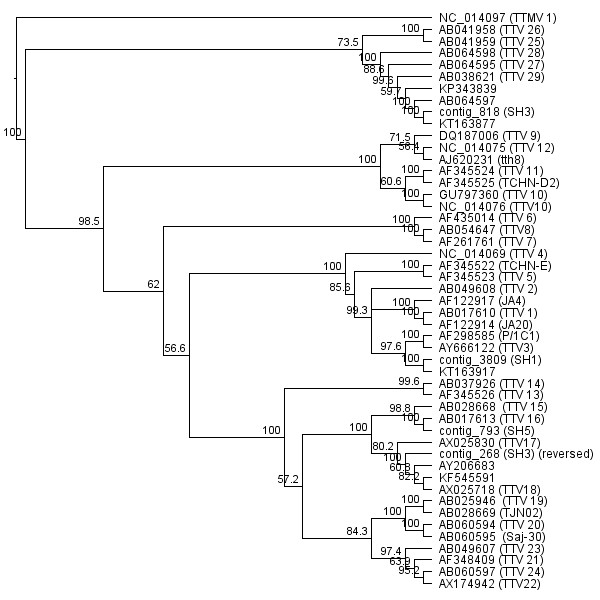
**

**Contig 1475**

**
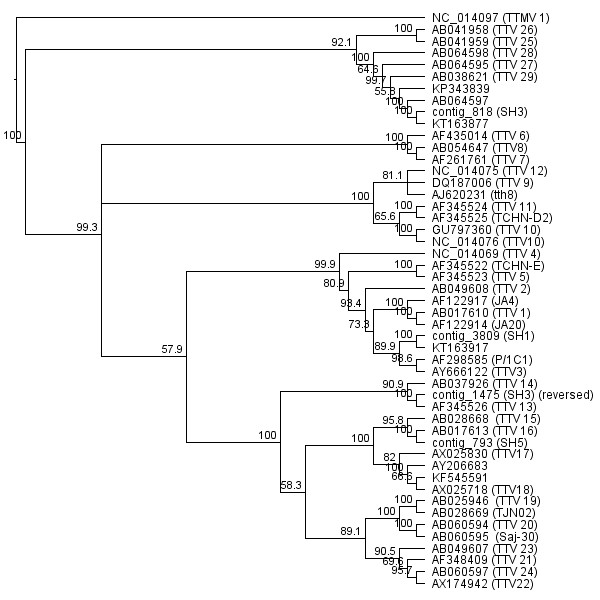
**

**Contig 1709**

**
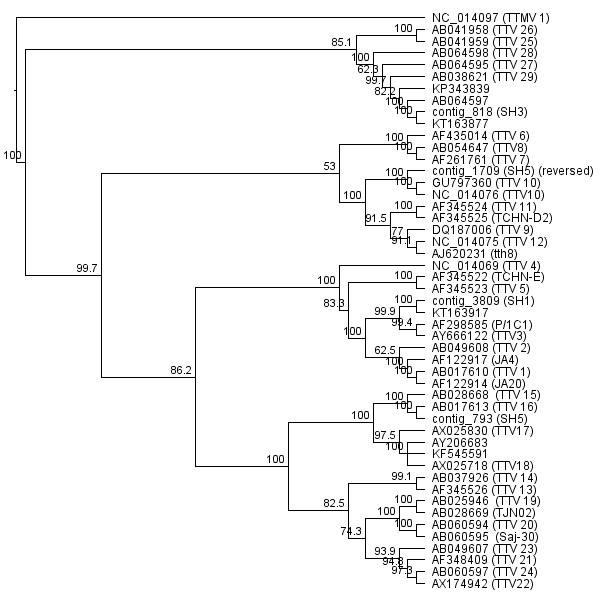
**

**Contig 2366**

**
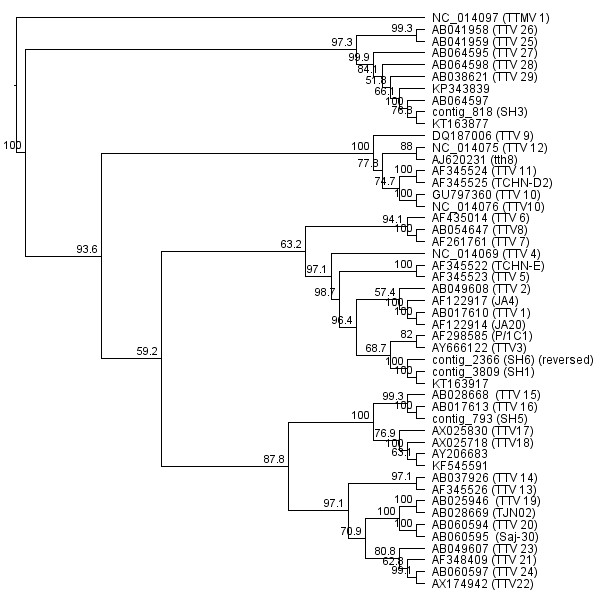
**

**Contig 2837**

**
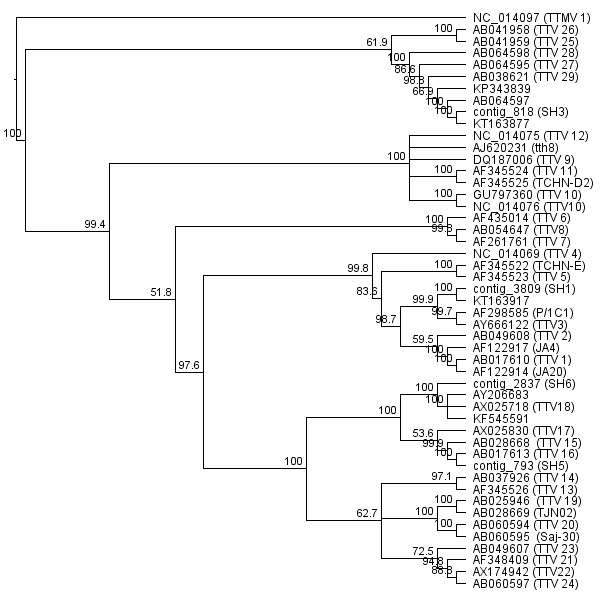
**

**Contig 5911**

**
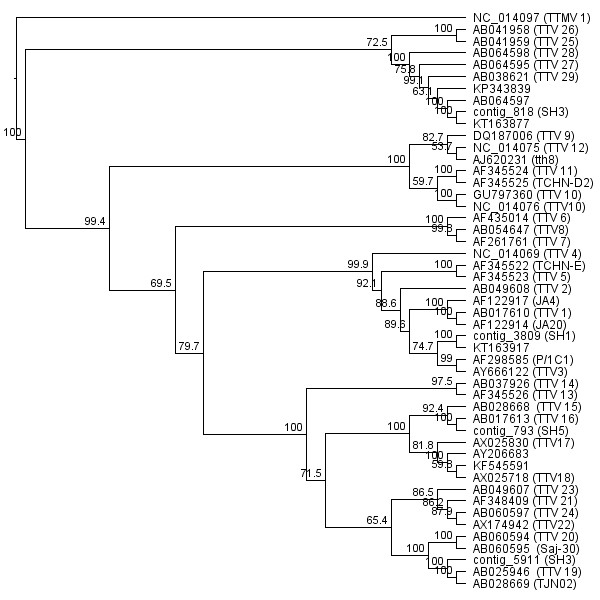
**

**Contig 6533**

**
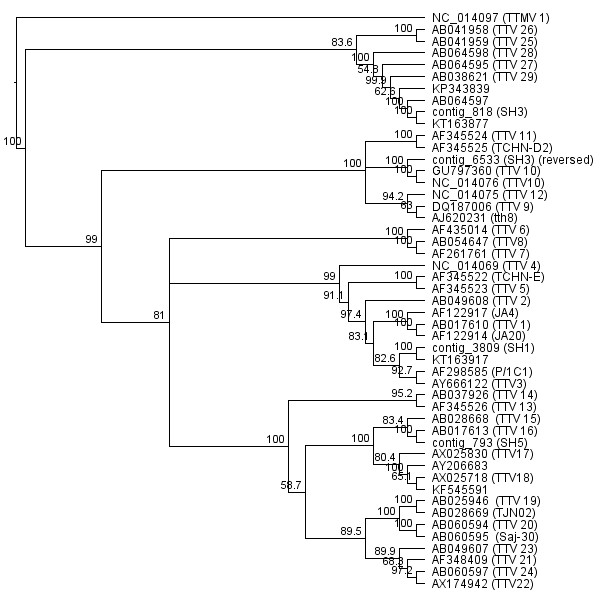
**

**Conti 7929**

**
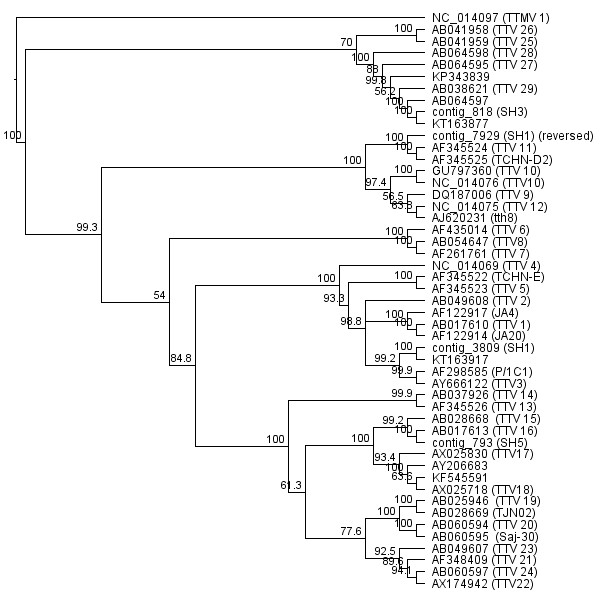
**

**Contig 9035**

**
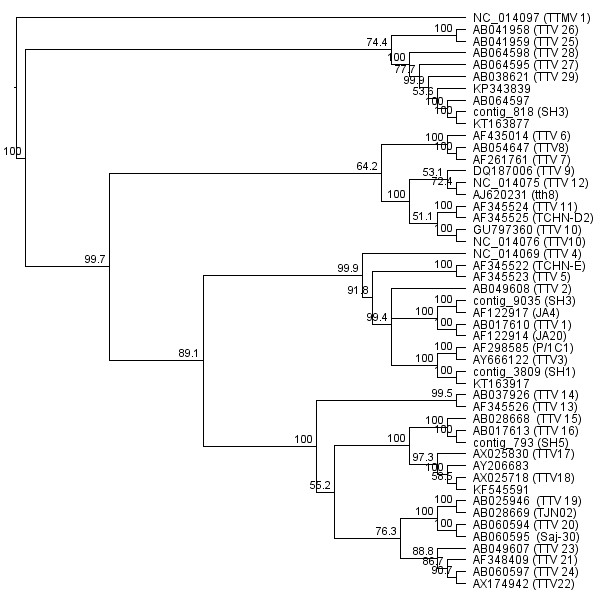
**

**TTMV**

**Contig 506**

**
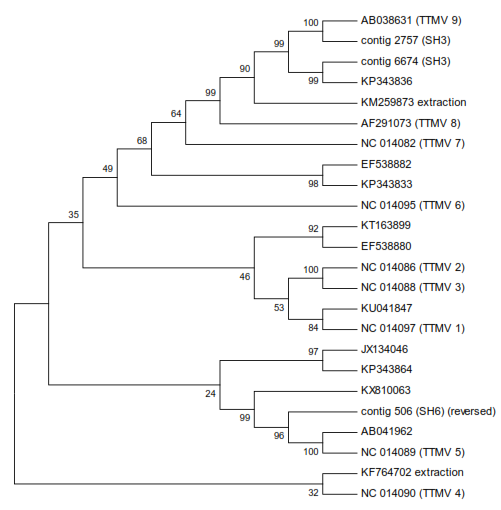
**

**Contig 2151**

**
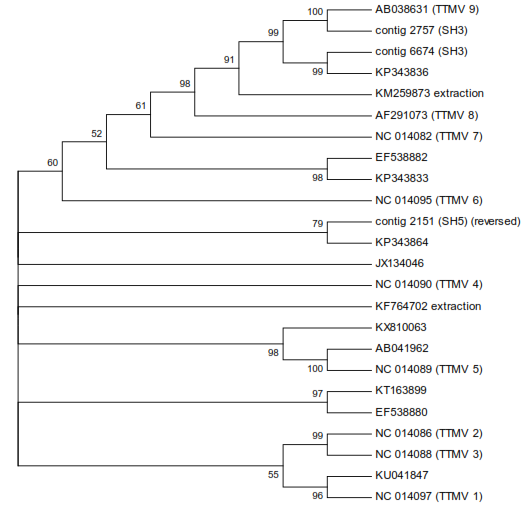
**

**TTMV Extended región (Letters in the original phylogenetic tree)**

**
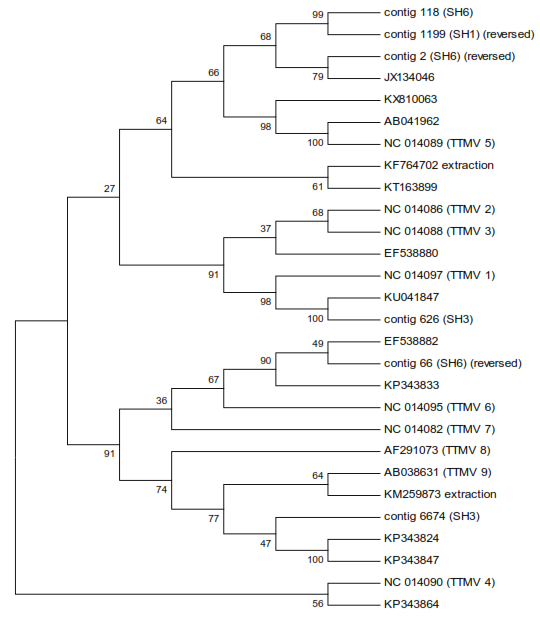
**

**TTMDV**

**Contig 1013**

**
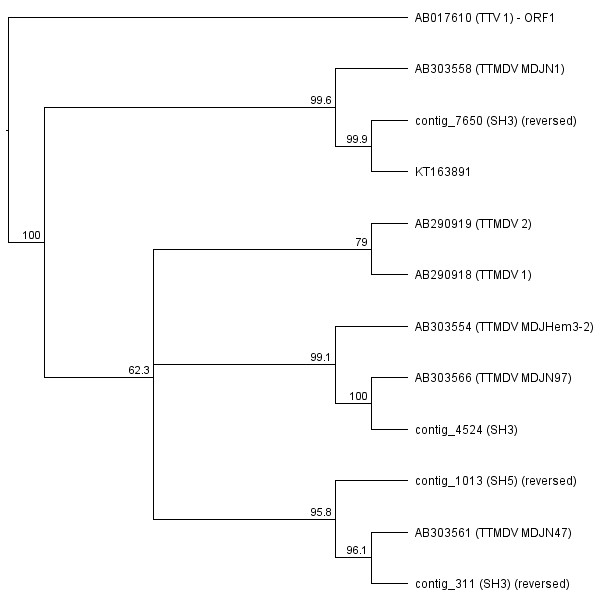
**

**Contig 1946**

**
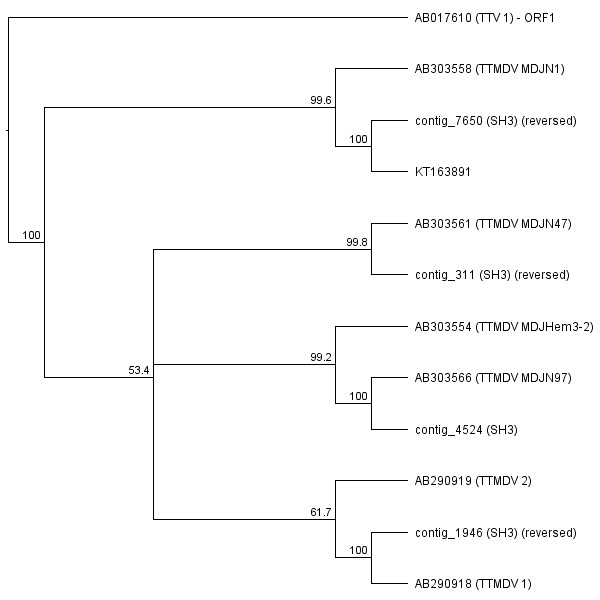
**

**Contig 16376**

**
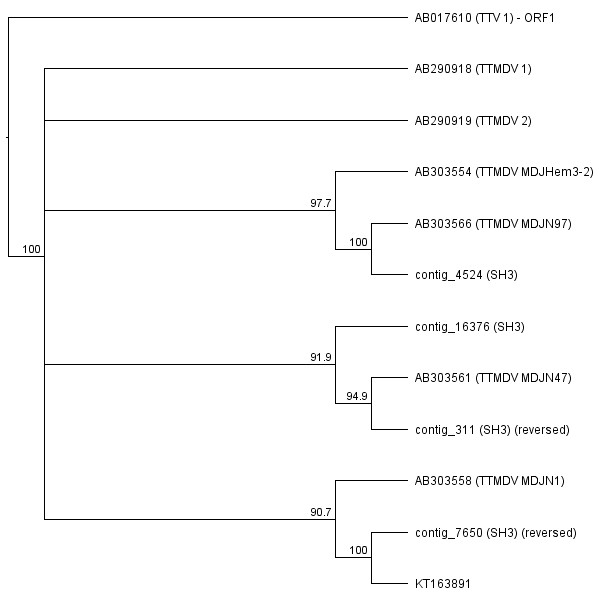
**

**New group Anello**

**COntig 1199 sh5**

**
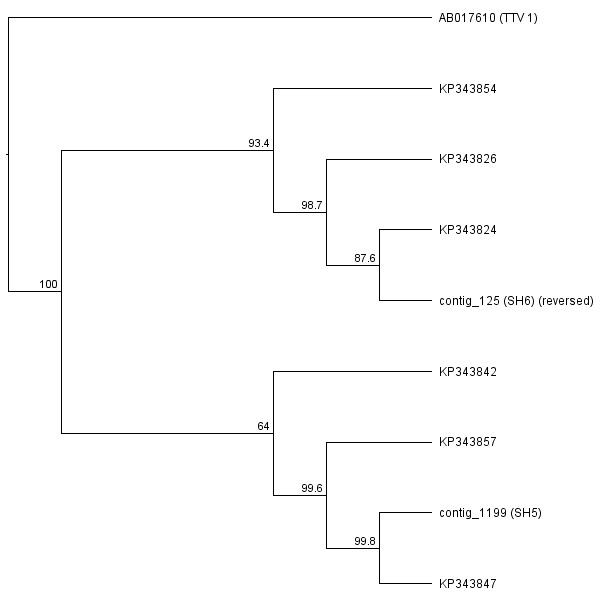
**
